# Supplementary material for: Association of epicardial fat with cardiac structure and function and cardiovascular outcomes: A protocol for systematic review and meta-analysis
Source: PLoS One. 2023 Apr 14;18(4):e0283482. doi: 10.1371/journal.pone.0283482 (PMC10104316; doi:10.1371/journal.pone.0283482)
Supplement: Attachment — Submitted filename: Response-PONE-D-22-25832_R1.doc [file pone.0283482.s002.doc]

The authors provide an interesting and potential important manuscript describing "Association of epicardial fat with cardiac structure and function and cardiovascular

outcomes: a protocol for systematic review and meta-analysis", The main issues concerning this paper are those concerning the potential associations between epicardial fat and cardiac structure and function and cardiovascular outcomes

There are some weak points that need to be addressed by the authors.

Minor

1. In general, excess adipocytes have some negative effects on cardiovascular disease, but adipocytes, as an endocrine cell, can also secrete molecules such as adiponectin, omentin, and others that are beneficial for cardiovascular conditions. Adipose secreted factors in the context of non obesity, may play a protective role against cardiovascular. Patients older than 18 years were selected in the text for the determination of epicardial fat volume using MRI or CT and were not qualitative about whether the selected subjects were obese or not. So it is recommended to add grouping in this respect.
2. On the other hand, the age factor has a direct effect on mortality and prognosis, in order to better represent the effect of epicardial fat on CVD, the age grouping can be further increased under the premise that the data collected in the articles are comprehensive.
